# Challenges of Anticoagulant Therapy in Atrial Fibrillation—Focus on Gastrointestinal Bleeding

**DOI:** 10.3390/ijms24086879

**Published:** 2023-04-07

**Authors:** Alina Scridon, Alkora Ioana Balan

**Affiliations:** 1 Physiology Department, University of Medicine, Pharmacy, Science and Technology “George Emil Palade” of Târgu Mureș, 540142 Târgu Mureș, Romania; 2 Emergency Institute for Cardiovascular Diseases and Transplantation of Târgu Mureș, 540136 Târgu Mureș, Romania

**Keywords:** atrial fibrillation, direct oral anticoagulants, gastrointestinal bleeding, thrombosis, vitamin K antagonists

## Abstract

The rising prevalence and the complexity of atrial fibrillation (AF) pose major clinical challenges. Stroke prevention is accompanied by non-negligible risks, making anticoagulant treatment an ongoing challenge for the clinician. Current guidelines recommend direct oral anticoagulants (DOACs) over warfarin for stroke prevention in most AF patients, mainly due to the ease of their use. However, assessing the bleeding risk in patients receiving oral anticoagulants remains—particularly in the case of DOACs—highly challenging. Using dose-adjusted warfarin increases threefold the risk of gastrointestinal bleeding (GIB). Although the overall bleeding risk appears to be lower, the use of DOACs has been associated with an increased risk of GIB compared to warfarin. Accurate bleeding (including GIB-specific) risk scores specific for DOACs remain to be developed. Until then, the assessment of bleeding risk factors remains the only available tool, although the extent to which each of these factors contributes to the risk of bleeding is unknown. In this paper, we aim to provide a comprehensive review of the bleeding risk associated with oral anticoagulant therapy in AF patients, with a highlight on the latest insights into GIB associated with oral anticoagulation; we emphasize questions that remain to be answered; and we identify hotspots for future research.

## 1. Introduction

With the continuous aging of the population and the increasing survival of patients with chronic cardiovascular diseases, atrial fibrillation (AF) has become the most common sustained cardiac arrhythmia encountered in clinical practice, affecting more than 43 million people worldwide [1]. Regardless of the presence of symptoms and the duration of the arrhythmia, the vast majority of AF patients require lifelong oral anticoagulation to prevent ischemic stroke. Although highly efficient, oral anticoagulation brings with it an increased risk of bleeding, particularly in certain categories of patients [1]. In this paper, we aim to provide a comprehensive review of the bleeding risk associated with oral anticoagulant therapy in AF, with a highlight on the latest insights into gastrointestinal bleeding (GIB) associated with oral anticoagulation.

## 2. Atrial Fibrillation—A Global Epidemic

The prevalence of AF has consistently increased over the past decades, positioning this arrhythmia as the most common sustained cardiac arrhythmia encountered in clinical practice [1]. Although the global prevalence of AF is estimated to be <1%, it increases to 3.7–4.2% in people aged 60–70 years, and to 10–17% in people aged ≥80 years [2,3]. Despite the major advancements in cardiovascular prevention strategies, AF prevalence is estimated to continue to increase in the following decades and to reach 10 million cases in the United States [4], 17 million in Europe [5], and up to 72 million in Asia [6] by 2050. Given the high morbidity, mortality, and considerable health care costs [6] associated with AF, this perspective represents a worrisome global epidemiological problem.

## 3. Ischemic Stroke—The Hidden Enemy of the Atrial Fibrillation Patient

One of the main concerns in AF patients is their increased risk of systemic and mainly cerebral embolism. This risk is present regardless of whether the arrhythmia is paroxysmal, persistent, or permanent [7]. In addition, patients with AF-related strokes have worse outcomes and more serious disability than those with strokes that are not related to AF [8].

All three components of Virchow’s triad (i.e., endothelial dysfunction, abnormal blood stasis, and altered hemostasis) contribute to intracardiac thrombus formation in AF patients, with hypercoagulant status being seen as the main culprit for the increased risk of stroke in this population [9]. Vascular and structural heart disease are compatible with Virchow’s criterion for endocardial dysfunction, blood stagnation in the left atrium meets the criterion for blood stasis, and abnormal blood constituents (e.g., abnormal procoagulant platelet factors) are compatible with the abnormal coagulation and fibrinolysis criterion [9,10].

**Figure 1:**
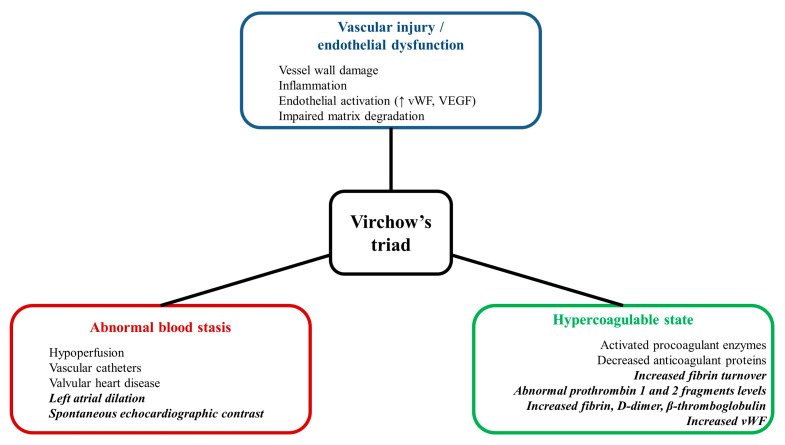
Virchow’s criteria as risk factors for ischemic stroke in the presence and absence of atrial fibrillation. Factors specific for the presence of atrial fibrillation are written in bold font. VEGF—vascular endothelial growth factor; vWF—von Willebrand factor. ↑ indicates an increase in biomarkers levels.

Many of the factors that contribute to thrombus formation in the fibrillating atria are well-established stroke risk factors, even in the absence of AF (Figure 1) [9,11,12]. Although AF has been associated with stroke even after adjustment for shared risk factors [9,11,12], there is no direct evidence that AF alone is sufficient to induce the occurrence of stroke. In fact, in AF patients that lack additional risk factors, the adjusted stroke rate appears to be essentially negligible [13]. This highlights the critical role of coexisting risk factors in stroke occurrence in the AF patient.

Indeed, several scores that incorporate various combinations of risk factors have been developed to stratify the risk of stroke in AF patients. Among these, the CHA_2_DS_2_-VASc score is currently the most widely used in clinical practice. More recently, the Age, Biomarkers, Clinical history (ABC)-stroke score, which includes age, cardiovascular biomarkers (i.e., NT-proBNP and high-sensitivity cardiac troponin T), and clinical history (i.e., prior stroke/transient ischemic attack), has been shown to outperform the CHA_2_DS_2_-VASc score in its ability to predict stroke in patients with AF and Congestive heart failure, Hypertension, Age ≥ 75 years, Diabetes mellitus, Stroke (doubled) (CHADS_2_) score ≥2 [14]. The GARFIELD-AF tool, which simultaneously calculates the risks of death, stroke, and bleeding, has also been shown to accurately predict stroke even in low-risk patients [15].

## 4. Stroke Prevention in Atrial Fibrillation

Despite the development of increasingly efficient arrhythmia detection strategies and of friendlier stroke prophylaxis regimens, the incidence of stroke remains high among AF patients, particularly if multiple risk factors are present [7]. Changes in guideline recommendations and the availability of newer, ‘friendlier’ drugs have led to a substantial increase in the proportion of AF patients that receive lifelong oral anticoagulation [16,17]. Throughout time, pharmacological strategies to prevent stroke in AF included antiplatelets, vitamin K antagonists (VKAs), and, over the past decade, direct oral anticoagulants (DOACs).

### 4.1. Antiplatelet Therapy—Less Effective than Oral Anticoagulation, but Just as ‘Bloody’

Due to its potent antithrombotic effects, aspirin, the most widely used antiplatelet agent, was an obvious candidate for stroke prevention in AF. Compared with placebo, aspirin therapy was associated with ≈20% reduction in the relative risk of stroke [18], and adding clopidogrel to aspirin further reduced this risk in patients with AF [19]. However, in large clinical trials, aspirin and even dual antiplatelet therapy were clearly outperformed by warfarin in their ability to prevent ischemic stroke [20,21]. Moreover, both single and dual antiplatelet therapies were associated with similar bleeding risk to that associated with warfarin [20,21]. The accumulating data thus placed antiplatelet agents as not only less efficient (by ≈40%) [22], but also as similarly hazardous as warfarin, leading to rapid and complete withdrawal of antiplatelet drugs as stroke prevention strategies in AF [23,24].

### 4.2. Warfarin—The ‘Blessed Poison’

With its ability to reduce the risk of stroke by more than two-thirds and mortality by one-quarter compared with control (aspirin or no therapy), warfarin is one of the most efficient prophylactic strategies that medicine has ever known [22]. Due to its wide availability, low cost, and easy-to-administer antidote, warfarin is widely used for stroke prevention in AF patients [22] and is the preferred oral anticoagulant in patients with mitral stenosis, valve replacement, or advanced renal disease [23]. However, its complex pharmacokinetics makes warfarin the target of numerous interactions. The manufacturer itself provides a list of over 200 drugs that can interfere with the anticoagulant effect of warfarin, and the list of food interactions is almost as generous [25]. Genetic polymorphisms, particularly in the CYP2C9 and VKORC1 genes, can also significantly affect the response of AF patients to warfarin therapy [26,27]. These interactions are even more important considering that the VKAs have narrow therapeutic window. Together, these factors make VKA anticoagulation extremely capricious and ‘sentence’ the AF patient to an endless stream of laboratory monitoring and dose adjustments. Meanwhile, the most optimistic statistics show that, even with extremely rigorous monitoring, patients receiving VKAs are adequately anticoagulated less than 70% of the time [28].

### 4.3. Direct Oral Anticoagulants—The ‘New Kids on the Block’

The numerous challenges associated with warfarin usage have emboldened researchers and pharmaceutical companies in their search for newer, ‘friendlier’ oral anticoagulants. To be considered ‘ideal’, an anticoagulant will have to be as effective as or even more effective than warfarin, and as safe as or even safer than warfarin. It will have to be available for oral administration, in fixed doses, have few food and drugs interactions, and have rapid onset and offset of action. It should have a predictable effect, without the need for monitoring, and it should have a widely available and safe antidote [29].

After more than 50 years of VKA monopoly, two classes of direct, non-VKA oral anticoagulants—direct thrombin inhibitors and direct factor Xa inhibitors—have been approved for stroke prevention in AF [23]. These new drugs are easier to administer, for both the doctor and the patient, and, due to their more stable pharmacokinetic and pharmacodynamic properties, shorter half-lives, fewer food and drug interactions, and larger therapeutic windows, they do not require routine monitoring or dose adjustments [29]. Large clinical trials have shown these agents to be non-inferior to warfarin in ischemic stroke prevention and to associate lower risk of fatal and intracranial hemorrhage than warfarin [30], which placed them as the preferred oral anticoagulants in the vast majority of AF patients [23,30]. Because of numerous reasons, however, the evaluation of anticoagulation status, at least in selected categories of DOAC-treated patients, is not entirely futile.

Dabigatran plasma levels have been shown to vary considerably with age, sex, bodyweight, kidney function [31], and, at least in experimental settings, with plasma lipid levels [32]. In turn, these varying levels have been shown to significantly affect both the thrombotic and the hemorrhagic risk of dabigatran-treated patients [31]. Important variations have also been reported for the direct factor Xa inhibitors, even in healthy individuals, although the clinical impact of such variations remains unknown to date [33,34]. Although DOACs have fewer interactions than the VKAs, they are also not free from interactions. The CYP3A4 enzyme, involved in factor Xa inhibitors metabolism, interacts with more than half of the commercially available drugs [35]. Many other drugs interfere with the P-glycoprotein, one of the main modulators of DOACs transport [36]. The implications of these interactions are extremely important considering that these proteins are the substrate of many cardioactive drugs that are widely used in AF patients, including digoxin, calcium channel blockers, and antiarrhythmic drugs [29]. The European Society of Cardiology has already issued a number of recommendations regarding such drug associations, and, for many other combinations, data are not yet available [36]. The anticoagulant effect of DOACs is also not immune to genetic variability. Polymorphisms in the CES1 gene have been shown to increase dabigatran plasma levels and to affect bleeding risk in these patients [37]. Genetic variations have also been shown to influence factor Xa inhibitors levels, although the polymorphisms identified to date do not appear to exhibit significant clinical impact [38,39].

## 5. Good Things Come with a Cost—Bleeding Risk in the Anticoagulated Atrial Fibrillation Patient

In the AF patient, oral anticoagulation is the sole therapy with proven benefits on survival [30,40]. Unfortunately, this benefit does not come without a cost. Altered sense of taste and gastrointestinal side effects (nausea, vomiting, abdominal pain, bloating, and flatulence) are not uncommon in patients undergoing warfarin therapy [41]. In certain patients, skin necrosis, purple toe syndrome, osteoporosis, calciphylaxis, and valvular and vascular calcification can also occur [41]. However, the single most common warfarin side effect is bleeding. When VKA anticoagulation is carefully monitored, the risk of major bleeding increased by ≈0.3% per year (from 1.0% in control to 1.3% in warfarin-treated patients), with an increase in the risk of intracranial hemorrhage by ≈0.2% per year (from 0.1% in control to 0.3% in patients treated with warfarin) [42].

All patients should therefore undergo thorough evaluation of their bleeding risk prior to and during oral anticoagulant therapy [23]. Numerous factors have been shown to increase the risk of bleeding in patients receiving anticoagulants, and several bleeding risk scores that incorporate various combinations of those factors (Table 1) have been developed in the attempt to reduce bleeding in this population. One of the most widely used bleeding risk scores is currently the HAS-BLED score [43,44]. Other scores, such as ABC, HEMORR_2_HAGES, or ATRIA, although have high specificity, have been shown to have only modest sensitivity [14]. Whereas certain factors (e.g., labile International Normalized Ratio [INR], anemia, malignancy, genetic background) are specific for different risk scores, others, such as advanced age, hypertension, or history of bleeding, are Incorporated in the vast majority of them [43,44]. Other factors, such as congestive heart failure or diabetes mellitus, although not generally included in the risk scores, have also been related to increased bleeding risk in different clinical studies [45]. In patients undergoing VKA therapy, the quality of anticoagulation, with a time in therapeutic range (TTR) > 70%, is regarded as a major player in the thrombotic/hemorrhagic risk balance [23,45]. Both an INR outside the recommended range and a TTR < 70% have been shown to increase the risk of bleeding, an INR > 3 being associated with a doubling in the incidence of major bleeding [45].

In their landmark clinical trials, most of the novel, non-VKA oral anticoagulants have been associated with lower risk of major bleeding, and all have shown significantly lower risk of intracranial hemorrhage compared to warfarin [46,47,48,49]. In a meta-analysis combining all four clinical trials, the use of DOACs was associated with similar rates of major bleeding, but with a significant, more than 50% reduction in the risk of intracranial hemorrhage compared with warfarin [30]. This does not mean, however, that DOACs are free of hemorrhagic risk. Despite their rapid onset and offset of action, more predictable pharmacodynamics, and fewer food–drug and drug–drug interactions, DOACs still associate a significant risk of major, including potentially life-threatening bleeding [50]. In different studies, fatal bleeding rates associated with the use of DOACs varied from 0.06% to 0.30%, while the rates of major bleeding ranged from 1.1% to 4.0% [50,51]. Data from the clinical trials and from real-life observational studies suggest that between-drug differences could also exist in regard to their safety. Whereas apixaban and low-dose (i.e., 110 mg bid) dabigatran appeared to reduce the risk of major bleeding compared to warfarin, this was not the case for rivaroxaban, nor for the high (i.e., 150 mg bid) dose of dabigatran [30,52]. However, given that no randomized clinical trial has performed so far a head-to-head comparison of the different DOACs, these data should not be used as a criterion for choosing between them.

All DOACs show, however, considerably better safety profiles than the VKAs, although there are certain considerations that temper the enthusiasm. In line with the suboptimal TTR achieved in the DOAC trials [30], bleeding rates in the warfarin groups were higher in those trials than those reported in the previous (warfarin vs. aspirin) trials [20]. The cutoff creatinine clearance of 30 mL/min utilized in the DOAC trials could also have influenced these results. Moreover, accumulating data clearly indicate that food and drug interactions, genetic variations, and phenotypic features can affect DOAC anticoagulation and can increase the bleeding risk in these patients [29]. Factors such as male sex, advanced age, hypertension, impaired renal function, diabetes, history of stroke or bleeding, anemia, and use of antiplatelet or anti-inflammatory drugs have all been associated with increased risk of major bleeding in AF patients treated with DOACs [53,54]. Patients with severe liver dysfunction are also at increased risk of bleeding secondary to coagulopathy. However, since patients with active liver disease were excluded from all DOAC trials, bleeding rates in these patients remain to date elusive [30]. Since all DOACs are partly eliminated via the kidneys, altered renal function can have a major impact on patients’ risk of bleeding. This is particularly the case for dabigatran, for which ≈80% of the dose is eliminated via the kidneys [55]. In a subgroup analysis of the Randomized Evaluation of Long-Term Anticoagulation Therapy with Dabigatran Etexilate (RE-LY) trial, patients with moderate kidney dysfunction had 2–3-fold higher dabigatran plasma concentrations and were more likely to develop bleeding complications than those with normal renal function [31]. Administration of DOACs without the need for monitoring could thus provide a false sense of safety, at least in certain categories of patients, in whom laboratory evaluation would seem desirable. Unfortunately, the laboratory techniques specific to these agents are not readily available in most laboratories, and, even if they were available, we do not know, for the moment, how effective DOAC anticoagulation should look like. And, even if we did, our options would still be extremely limited. If, for the VKAs, an INR outside the therapeutic window is followed by dose adjustment, such a possibility does not exist for the DOACs, for which only specific doses have been approved, and not the drugs per se.

Careful selection of patients for DOAC treatment is therefore essential, and thorough bleeding risk evaluation should be performed in all candidates for DOAC therapy prior to and during anticoagulation [23]. A score comprising age, history of bleeding, and non-bleeding-related hospitalizations within the last 12 months has been proposed by Rutherford et al. to assess bleeding risk in AF patients treated with DOACs [56]. Other scores have also been developed to assess the risk of bleeding in patients receiving anticoagulants, but not all have been validated in patients undergoing DOAC therapy. Among those evaluated for validation, the HAS-BLED, HEMORR_2_HAGES, RIETE, and CHEST scores did not seem to reach good diagnostic accuracy for predicting bleeding in patients receiving DOAC therapy [57].

**Figure 2:**
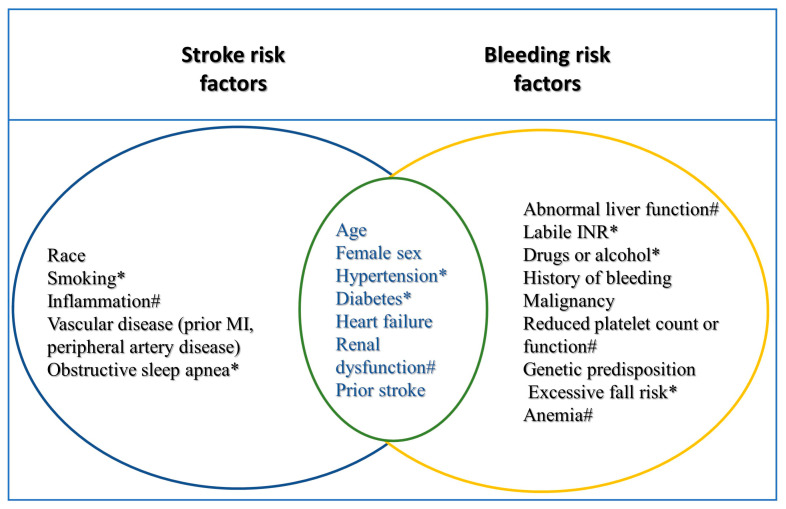
Risk factors for stroke, bleeding, and shared risk factors in patients with atrial fibrillation. *—indicates modifiable bleeding risk factors. #—indicates potentially modifiable bleeding risk factors. INR—International Normalized Ratio; MI—myocardial infarction.

In addition, the fact that many of the factors known to increase patients’ propensity to thrombosis also increase their bleeding risk (Figure 2) further complicates the management of anticoagulant therapy. One should keep in mind, however, that, although bleeding is a major concern in anticoagulated patients, the risk of thrombosis most often exceeds that of bleeding, including in the elderly, in those with cognitive impairment, or those with frequent falls [23]. Bleeding risk scores should therefore be used to identify and correct modifiable bleeding risk factors [23] and not as a pretext for abstention from oral anticoagulation.

## 6. Gastrointestinal Bleeding in the Anticoagulated Atrial Fibrillation Patient

Bleeding risk scores have been developed to allow the estimation of major bleeding risk. However, none of them allows differentiation of the type of hemorrhage [44], and, although it has been suggested that the HAS-BLED score can be used to estimate GIB risk in VKA-treated patients [43], scores specific for GIB risk have not been developed to date.

### 6.1. Gastrointestinal Bleeding in Patients Treated with Vitamin K Antagonists

Intracranial hemorrhage is the most feared anticoagulation-related complication. However, bleeding in the digestive tract is by far the most common type of hemorrhage in VKA-treated patients, leading to significant increase in hospitalization rates and important resource utilization [58,59]. Thirty-day mortality rates of up to 15.5% have been reported in patients with VKA-related GIB, although this high mortality is likely to be mainly due to comorbidities, rather than to VKA therapy per se [60]. In the study by Coleman et al., GIB incidence in patients treated with warfarin was 3.9% per patient-year, three times higher than in the general population [61]. In patients with a history of GIB, resumption of warfarin therapy resulted in recurrence of bleeding in 27.3% of cases [61]. The upper digestive tract appears to be the most common site for bleeding in patients receiving VKA therapy (8–15%), while hemorrhages in the lower digestive tract are found in ≈7% of patients [62].

Factors such as advanced age, increased INR, history of GIB, and liver cirrhosis have all been identified as independent predictors of GIB in warfarin-treated patients [58]. Previous history of gastrointestinal ulcer has also been related to a more than 6-fold increase in the risk of GIB [62]. The use of nonsteroidal anti-inflammatory drugs (NSAIDs) is well recognized for its negative effects on GIB risk [62]. Interestingly, however, not all studies have reported a significant difference in NSAIDs usage between patients with and without GIB [63].

When GIB does occur, the risk–benefit ratio of oral anticoagulation should guide the clinician’s decision to continue, halt, or reverse the effects of VKA therapy [64]. The clinical severity of the hemorrhage, the INR value, the duration of a potential endoscopic hemostatic procedure (using adrenaline injection, argon plasma coagulation, endoscopic hemoclips, unipolar or bipolar electrocoagulation, or, more rarely, sclerotherapy), and the thrombotic risk of the patient must be taken into account in the decision-making process [64,65]. Reversal of VKA effects is not urgent if GIB is self-limited GIB [64,65]. In patients with clinically significant GIB and supratherapeutic INR in whom the life-threatening risk of continuous bleeding outweighs the risk of thrombosis, discontinuation of anticoagulant therapy is justified [64]. To decrease the INR before urgent endoscopy, VKA reversal can be obtained using vitamin K, fresh frozen plasma (FFP), prothrombin complex concentrates (PCC), or recombinant activated factor VIIa (rFVIIa) [64]. No target INR value has been established so far for safe endoscopic therapy. However, obtaining an INR of 1.5–2.5 by VKA reversal appears to be effective for endoscopic therapy [66]. Data regarding the benefit of early correction of VKA-associated coagulopathy on clinical outcomes in patients undergoing endoscopic therapy is limited. Warfarin therapy at presentation does not appear to have a negative impact on bleeding-related mortality, and the INR value measured prior to endoscopic treatment did not correlate with the need for transfusion, duration of hospitalization, or mortality [67]. Moreover, in patients in whom the INR was in the therapeutic range, rebleeding rates did not differ substantially compared with those in whom the INR was supratherapeutic (i.e., ≥4) [67]. However, since coagulopathy was identified as the strongest clinical predictor of failed endoscopic hemostasis and data on patients without VKA reversal have not been reported to date, experts recommend that endoscopy be delayed until an INR value of 2.5 is achieved [65].

In order to reduce the INR before urgent endoscopy, vitamin K, FFP, PCC, and rFVIIa can be used [68]. Vitamin K, by promoting the synthesis by the liver of new functional coagulation factors II, VII, IX, and X, lowers the INR value in 2–4 h after administration of 5–10 mg in slow IV infusion (minimum 30 min) [68]. Given that the effect occurs late, vitamin K is not ideal for emergency reversal of anticoagulation [59,68]. However, its administration provides a sustained correction of coagulopathy that extends beyond the correction provided by FFP and PCC. Another method for VKA reversal is the use of FFP, consisting of the fluid portion of human blood containing vitamin-K-dependent clotting factors [59,68]. The recommended dose is an IV infusion of 15 mL/kg (approximately 3–4 units of 250 mL plasma for an adult weighing 70 kg). A more rapid correction of the INR can be obtained with PCCs, which are pharmacological products containing inactivated concentrates of factors II, IX, and X, and varying amounts of factor VII [59,68]. These are administered according to their factor IX content and initial INR, usually at a dose of 25–50 IU of factor IX/kg. Although not licensed for VKA reversal, rapid correction of elevated INR values and treatment of VKA-associated bleeding using rFVIIa has been described in case reports and small case series [59,68]. However, its routine use should be avoided until sufficient evidence to attest its effectiveness and safety is available.

### 6.2. Gastrointestinal Bleeding in Patients Treated with Direct Oral Anticoagulants

Based on the results of large clinical trials and observational real-life registries, a consensus seems to have been reached regarding the lower overall risk of bleeding associated with DOACs compared with warfarin. Intriguingly, however, this does not seem to apply to GIB. In fact, the use of DOACs was associated with a 25% higher risk of GIB than warfarin in the clinical trials. Both major and minor GIB were included in that analysis, and the results seem to have been mainly driven by the occurrence of minor hemorrhages [69]. Furthermore, not all DOACs and not all DOAC doses have been associated with higher risk of GIB compared with warfarin. While the 150 mg dose of dabigatran has been shown to increase the risk of GIB, the 110 mg dose showed a risk similar to that of VKA therapy [46]. Apixaban did not show significant differences in the risk of major GIB compared with warfarin, while non-major bleedings, including non-major GIB, were significantly lower in the apixaban group [30,53]. Edoxaban 60 mg daily was associated with increased major GIB rate compared with warfarin [47]. The 30 mg dose showed, however, lower rates of GIB than warfarin [47]. In a population-based study comparing bleeding rates for dabigatran, rivaroxaban, and apixaban, apixaban showed the best safety profile for GIB compared with both rivaroxaban and dabigatran, and rivaroxaban appeared to have a less favorable safety profile than dabigatran [70]. Similar results were also reported in a meta-analysis of 43 randomized clinical trials [69]. Differences in the site of hemorrhage have also been reported between certain DOACs and the VKAs. In the case of dabigatran, the upper and lower parts of the gastrointestinal tract appeared to be similarly affected (i.e., 53% of GIB occurred in the upper, and 47% in the lower gastrointestinal tract), whereas, in the warfarin-treated patients, three-quarters of GIBs occurred in the upper gastrointestinal tract [71].

No definitive mechanism has been proposed to date to explain the increased risk of GIB associated with DOAC compared with VKA use, or the differences that appear to exist between DOACs [72]. In the case of dabigatran, the tartaric acid coating, which has caustic effect on the intestinal mucosa, could provide at least partial explanation. Incomplete absorption of DOACs into the intestinal mucosa may also lead to a local anticoagulant effect. For rivaroxaban, administration in a single dose may lead to greater variance in drug plasma concentration, and thus to increased GIB risk.

Similarly to what was seen in VKA-treated patients, advanced age, particularly >75 years, has also been associated with increased GIB risk in DOAC-treated patients, and Chinese patients appear to be more prone to GIB than non-Asian individuals [73]. Due to drug accumulation, renal dysfunction has also been associated with increased risk of GIB in DOAC- and particularly in dabigatran-treated patients [69]. Patients with severe hepatic impairment have been excluded from the clinical trials. Data on GIB risk in DOAC-treated patients with liver disease are therefore extremely limited. Recent studies do not appear to indicate, however, a significant increase in bleeding in patients with advanced liver disease undergoing DOAC therapy [72,74]. Other factors, such as the use of gastrotoxic agents, alcohol consumption, and Helicobacter Pylori infection, have also been associated with increased GIB risk in DOAC-treated patients [75]. In the absence of specific GIB risk scores, all these risk factors should be considered when initiating chronic DOAC therapy. The presence of colonic diverticulosis, angiodysplasia, and history of peptic ulcer should also alert the clinician and all modifiable bleeding risk factors should be identified and corrected prior to initiation of anticoagulant therapy [72].

If bleeding does occur in DOAC-treated patients, discontinuation of treatment may be considered only after weighing the risks and benefits [23,76]. Given the short half-lives of DOACs, the risk of thrombotic events increases substantially even if they are stopped for a short period [23]. Stopping anticoagulation is therefore not recommended if bleeding is minimal [23]. If halting DOAC therapy is required, DOAC plasma levels return to normal within 12–24 h [76]. To prevent further absorption, particularly in the case of a recent dabigatran overdose, gastric lavage and oral charcoal may be considered if DOACs have been ingested within the last 2–3 h [77]. Non-specific pro-hemostatic agents such as activated and non-activated PCCs and rFVIIa have not been studied in large studies. However, in a few small case series, bleeding has been effectively controlled with PCC and, as a result, it is indicated in major hemorrhages [78]. On the other hand, there is no place for the use of vitamin K or FFP as antidotes against DOACs. Other, non-specific strategies, such as antifibrinolytics (tranexamic acid, epsilon-aminocaproic acid), and desmopressin can also be used in DOAC-treated patients with bleeding [76]. Specific DOAC reversal agents (i.e., idarucizumab for dabigatran; andexanet alfa for factor Xa inhibitors) are also available [23]. Idarucizumab (5 g IV) is only recommended if the use of dabigatran is certain and if thrombin time is prolonged [76]. When using andexanet (low-dose protocol—400 mg dose given at a rate of 30 mg/min, followed by an infusion of 4 mg/min for up to 2 h; high-dose protocol—800 mg administered at a rate of 30 mg/min, followed by an infusion of 8 mg/min for up to 2 h), clinicians should also consider its prothrombotic potential and perform careful monitoring [76]. Hemodialysis and hemoperfusion may be useful in patients treated with dabigatran, but not with factor Xa inhibitors, which are highly protein-bound [76].

Methods specific to GIB can also be applied, regardless if bleeding is VKA- or DOAC-related. Patients with GIB undergoing anticoagulation therapy should be evaluated endoscopically and the timing until endoscopy should be decided according to the severity of the hemorrhage [65,72]. Whenever possible, postponing the endoscopic procedure can improve detection of bleeding and facilitate endoscopic therapy [65]. Hemodynamically unstable patients, however, should undergo rapid, symptoms-guided endoscopic evaluation [65,72]. In the case of endoscopic treatment failure, radiological and surgical interventions can be used as alternatives [79].

### 6.3. Resuming Anticoagulation after Major Gastrointestinal Bleeding

In the case of GIB occurrence, temporary discontinuation of anticoagulant therapy is often required to reduce the risk of bleeding. However, permanent discontinuation of anticoagulant therapy may increase the risk of stroke and death in patients with AF. Therefore, it is important to consider the risks and benefits of both continuing and discontinuing anticoagulant therapy in patients with AF who have experienced an episode of GIB. Masiero et al. evaluated over 11,000 patients with AF and GIB and found that temporary discontinuation of anticoagulants was not associated with an increased risk of stroke or death within 30 days [80]. However, in a study evaluating more than 3000 patients with AF and GIB, temporary discontinuation of anticoagulants was associated with an increased risk of stroke and death 90 days after the bleeding event. In addition, continued anticoagulant therapy was not associated with increased risk of recurrent bleeding [81], and restarting oral anticoagulation was associated with lower risk of all-cause mortality and thromboembolic events [82]. There is limited evidence regarding the ideal timing of resuming anticoagulant therapy. The optimal timing for restarting anticoagulant therapy may vary depending on the severity of GIB and individual risk of thromboembolic events. In the study by Qureshi et al., restarting therapy 7–30 days after GIB was associated with a decreased risk of thromboembolic events and mortality without an increased risk of recurrent GIB [82].

The advantages and disadvantages of restarting VKA, as well as the appropriate timing to resume VKA therapy in patients with GIB, have not been sufficiently evaluated. The risk of recurrent GIB was significantly greater if warfarin was resumed within the first week of major GIB [83]. The cause of the bleeding dictates the probability of rebleeding after successful hemostasis. However, risk factors for GIB must be assessed and corrected before restarting VKA treatment. In a study by Chen et al., 27.3% of patients who resumed warfarin therapy experienced recurrent GIB, while thromboembolic events were seen in 16.7% of patients who did not continue warfarin therapy [58]. A warfarin dose adjusted to maintain an INR of 2.0 or less could be an alternative anticoagulation strategy to prevent thromboembolic events while minimizing the risk of GIB. Long-term acid suppressants may also be considered, especially in patients with peptic ulcer bleeding [84].

The timing for restarting anticoagulant therapy with DOACs was not evaluated. However, considering that restarting oral anticoagulation among patients with AF and GIB was associated with lower risk of all-cause mortality and thromboembolism, these agents should be started as soon as possible after weighing the hemorrhagic risk [82]. There are studies suggesting that restarting DOACs after a GIB episode may be safe and effective in preventing stroke in patients with AF. Chan et al. showed that the risk of GIB recurrence was similar in patients who received DOACs, compared to those in whom anticoagulant therapy was stopped [85]. However, it should be kept in mind that there is no standardized approach for restarting DOACs after a GIB episode and that the decision must be individualized for each patient. Dose reduction could be considered for patients with an increased risk of GIB recurrence. Moreover, changing the DOAC to another one with a lower risk of GIB could be an option.

## 7. Clinical Implications

Studies have shown that AF patients receiving anticoagulant therapy are at increased risk of GIB. Identification of risk factors and use of existing risk scores, although not specific for GIB, can help prevent hemorrhages through appropriate monitoring and management of patients. Identifying patients who are most susceptible to GIB contributes to an appropriate management of anticoagulant therapy to minimize risk. Anticoagulant therapy may need to be adjusted to reduce the risk of bleeding. Once GIB occurs, identifying the best bleeding control strategies are necessary to minimize the risk of death. Continuous evaluation of the effectiveness of different therapeutic options for GIB in patients with AF will lead to improved treatment and patient outcomes and contribute to the development of updated clinical guidelines for the management of AF and GIB.

## 8. Gaps in Evidence and Future Research

Despite the numerous clinical trials and observational studies conducted to date, numerous questions remain regarding the risk of bleeding associated with anticoagulant and particularly with DOAC therapy. The lack of easily available laboratory parameters to show the efficacy and safety of DOAC therapy makes it difficult, if not impossible, to detect inappropriate anticoagulation, which is likely to remain unnoticed until a thrombotic or bleeding complication occurs [86,87]. Monitoring the effects of DOACs would certainly be useful at least in specific clinical settings, such as suspicion of overdose, acute thrombotic or bleeding events, surgery, or acute renal failure [88]. Although activated partial thromboplastin and prothrombin time may be used to estimate anticoagulation with dabigatran or certain factor Xa inhibitors, both tests provide only qualitative information about the presence (but not the absence) of the drug [88]. More complex laboratory tests, such as the diluted thrombin time and the ecarin chromogenic assay, for dabigatran, and the chromogenic anti-factor Xa assays, for the Xa inhibitors, have been developed. However, none of them are routinely available in clinical practice. Development of widely available, easy-to-use tools to evaluate DOACs in various clinical settings is therefore imperative.

Accurate bleeding (including GIB-specific) risk scores for DOAC-treated patients also remain to be developed. Until then, the assessment of proven risk factors for bleeding is the only available tool, although the extent to which each of these factors contributes to the risk of bleeding remains unknown to date. Finally, the need for DOAC dose adjustment, at least in selected categories of patients, remains a pending issue [23]. Despite their more stable pharmacokinetics compared to warfarin, concerns exist that the ‘one dose fits for all’ strategy may not be entirely appropriate [89]. Plasma concentrations of DOACs have been shown to be affected by parameters such as drug interactions, renal impairment, or bodyweight, and evidence on the optimal dose of DOACs in ‘sensitive’ categories of patients is still lacking [89].

## 9. Conclusions

The prevalence of AF is constantly increasing globally, making AF an epidemic condition associated with increased morbidity and mortality. The increased risk of stroke associated with this arrhythmia is considerably reduced by chronic oral anticoagulant therapy. The advent of DOACs has changed the landscape of oral anticoagulation and has reduced the risk of major and intracranial hemorrhage compared to warfarin. Intriguingly, however, this does not seem to apply to the risk of GIB. Numerous other questions also remain regarding the risk of bleeding associated with DOAC therapy. The assessment of proven risk factors in DOAC-treated patients is essential, but the extent to which each factor contributes to the global risk of bleeding remains unknown to date. Given the expanding usage of DOACs, future studies will have to clarify these issues.

## Figures and Tables

**Table 1:** Most common bleeding risk scores used in clinical practice for hemorrhagic risk assessment in patients with atrial fibrillation undergoing oral anticoagulation.

	Bleeding Risk Scores			
	HAS-BLED	ABC	HEMORR_2_HAGES	ATRIA
**Age**	>65 years	Age	>75 years	≥75 years
**Biomarkers**	Labile INR	Hemoglobin Troponin T Growth differentiation factor-15	Reduced platelet count or altered platelet function Anemia	Anemia
**Clinical history**	Hypertension Abnormal kidney and liver function Stroke Bleeding Drugs Alcohol	History of bleeding	Liver or kidney disease Alcohol abuse Malignancy Bleeding Genetic factors Excessive fall risk Stroke	Bleeding Severe renal disease Hypertension
**Maximum score**	**9 points**	**>15%**	**12 points**	**10 points**

INR—International Normalized Ratio.

## Data Availability

Not applicable.
